# The Effect of Post-Reproductive Lifespan on the Fixation Probability of Beneficial Mutations

**DOI:** 10.1371/journal.pone.0133820

**Published:** 2015-07-31

**Authors:** Stefano Giaimo, Annette Baudisch

**Affiliations:** Max Planck Research Group: Modeling the Evolution of Aging, Max Planck Institute for Demographic Research, Rostock, Germany; University of Massachusetts, UNITED STATES

## Abstract

Post-reproductive lifespan is a common trait among mammals and is usually considered to be neutral; i.e. with no influence on population dynamics. Here, we explore the role of post-reproductive lifespan in the fixation probability of beneficial genetic variation. We compare two separate, stationary populations living in a constant environment that are equivalent except for the average time their respective members spend in the post-reproductive stage of life. Using a recently derived approximation, we show that fixation of a beneficial mutation is more likely in the population with greater post-reproductive longevity. This finding is surprising, as the population with more prolonged post-reproductive lifespan has smaller effective size and the classic population-genetic model would suggest that decreasing effective size reduces fixation chances of beneficial mutations. Yet, as we explain, in the age-structured case, when effective size gets smaller because of longer post-reproductive lifespan but census size is kept equal, a beneficial mutation has a higher likelihood to get fixed because it finds itself at higher initial frequency.

## Introduction

In populations with age structure, the fate of genetic variability can be influenced by life history features [1–10]. Post-reproductive lifespan is a life history trait that is typical of some mammals [11], and is especially developed in humans who can live for decades after having given birth to their last offspring. To our knowledge, the role that survival beyond the last reproductive age may play in population genetics models of adaptive evolution has not been investigated yet. This is probably because models with ideally infinite census size are insensitive to the presence of post-reproductive individuals [12]. The sole exception—which we will not consider in the present work—is when social interactions in the population are postulated to generate correlations between the survival of individuals that are past their last reproductive age (i.e. grandmothers) and the reproductive success of their still-reproducing relatives (i.e. daughters and granddaughters) [13, 14]. However, it is well known that post-reproductive lifespan reduces the population effective size [15, 16], which is a parameter inversely proportional to the genetic change in a finite population due to random sampling of gametes through generations [17]; i.e. drift. The classic model for populations without age structure suggests that a reduction in effective size goes to the detriment of fixation chances of a beneficial mutation [18, 19]. Hence, one would intuitively be led to infer that a more prolonged post-reproductive lifespan slows adaptive evolution in a population. In the present paper, we will show that this intuition is wrong and explain why it is so. Here is how we will proceed. We will describe the demography of two separate populations that are equivalent except for the average time their respective members spend in the post-reproductive stage of life. Then, we will introduce the classic model of Kimura [19] for the fixation probability of a beneficial mutation in a population without age structure, and the corresponding model proposed by Vindenes et al. [3] for a population with age structure. Using the latter, we will establish in which of the two previously described populations a mutation with some beneficial effect is more likely to go to fixation. In the light of the obtained results, we will discuss the relationship between Kimura’s model and the model with age structure. To keep our treatment as simple as possible, we consider asexual haploid populations. However, as we will suggest, our results should extend to the sexual diploid case.

## Two Populations with Different Post-Reproductive Longevity

Consider two separate, monomorphic populations *A* and *B* that have age structure and are composed of asexual haploids. Suppose both populations live in a constant environment, grow in a density independent manner, and are of size *N*. We observe these two populations at discrete time points just before the breeding season. We assume demographic stability for both populations; that is, in each population, the fraction, *u*_*k*_, of individuals in some age class *k* is constant over time. The life table in *A* is identical to the life table in *B* up to the last reproductive age *ω*. But life expectancy at the age of last reproduction, *e*_*ω*_, is different between *A* and *B*. It is a classic result in life history theory that these two populations must have the same multiplicative growth rate, *λ*, from one time period to the next, as *λ* is insensitive to the presence of post-reproductive individuals when survival and reproduction at pre-reproductive and reproductive ages do not depend on post-reproductive survival [20]. We make the assumption that *A* and *B* are stationary; that is, *λ* = 1. Following Fisher and Charlesworth [12] [21], we define the reproductive value,*V*_*k*_, of an individual in some age class *k* to be the present value (i.e. accounting for population growth) of the offspring that this individual is still expected to produce. Formally, $V_{k}=\frac{\lambda^{k}}{l_{k}}\sum_{x=k}^{\omega }l_{x}m_{x}\lambda^{-x}$, where *l*_*x*_ is the probability that a newborn survives to age *x*, and *m*_*x*_ is the number of newborns produced by an individual of age *x*. Under the assumption of stationarity, offspring produced at different time points in the future have the same present value, as they equally contribute to the population given the constant size of this. Therefore, when *λ* = 1, *V*_*k*_ is simply the expected future number of offspring to be produced by an individual of age *k*. Evidently, all individuals that are not post-reproductive have nonzero reproductive value, while post-reproductive individuals have zero reproductive value. Given that *A* and *B* have identical life tables up to *ω*, an individual of a given age in *A* must have the same reproductive value as an individual of the same age in *B*. The mean age at motherhood, *T*, in the population is also shared by *A* and *B*, because it is a quantity calculated over characteristics the individuals possess prior to reproductive cessation. However, the birth rate *b* must differ between *A* and *B*. In a stationary population, the birth rate must equal the death rate. As post-reproductive longevity is more prolonged in one of the two populations, the death rate in this same population must be lower. To retain stationarity, the birth rate must also be lower. Assume that

$$e_{\omega ,A}>e_{\omega ,B}.$$ (1)

This means that life expectancy at the last reproductive age is greater in *A* than in *B*. Using again subscripts to indicate the population to which the value of a given demographic quantity refers, we can write

$$\lambda_{A}=\lambda_{B}=1,$$ (2.1)

$$N_{A}=N_{B}=N$$ (2.2)

$$V_{k,A}=V_{k,B}=V_{k},\;\;\forall k,$$ (2.3)

$$T_{A}=T_{B}=T,$$ (2.4)

$$b_{A}<b_{B}.$$ (2.5)

These quantities will turn out useful below to compare the ability of the two populations to retain novel genetic variants that are advantageous.

## Fixation of a Beneficial Mutation

### Model without age structure

Consider a monomorphic haploid population of asexual organisms without age structure that lives in a constant environment and has fixed census size *N*. To keep size constant, each individual in the population has an average fecundity of unity. Suppose that a single mutant is initially present with average fecundity (1 + *s*) where *s* > 0. As shown by Kimura [19], the probability that the mutation ultimately gets fixed is approximately

$$s\frac{N_{e}}{N},$$ (3)

provided that *N* and *s* are not too small. Here *N*_*e*_ is the effective size of the population. The model says that the probability of fixation of the mutation, which is initially found as a single copy, increases with the beneficial effect of the mutation, and decreases with genetic drift and initial census size.

### Model with age structure

Consider a demographically stable, monomorphic population with age structure. The population is stationary (*λ* = 1), lives in a constant environment, and is not subject to density dependence. Suppose that a single mutant of age *k* is initially present. Assume that a hypothetical, separate population exclusively comprised of mutants of this kind would grow at a multiplicative rate (1 + *s*) where *s* > 0. However, *s* should be sufficiently small that there is a negligible difference between the reproductive value of the mutant, $V_{k}^{*}$, and the reproductive value of a resident individual of the same age as the mutant, $V_{k}\approx V_{k}^{*}$. Under these assumptions, Vindenes et al. [3] showed that the fixation probability of the mutation, which is initially found as a single copy, is approximately

$$\frac{1-\text{exp}\left\{-2s\frac{v_{k}}{N}\frac{N}{\sigma^{2}}\right\}}{1-\text{exp}\left\{-2s\frac{N}{\sigma^{2}}\right\}},$$ (4)

Where *v*_*k*_ is the reproductive value of a resident individual of the same age as the initial mutant relative to the average reproductive value in the stable resident population. As the average reproductive value in the stable population, Σ *u*_*i*_ *V*_*i*_, equals the product of the birth rate and the mean age at motherhood [15], we can write

$$v_{k}=\frac{V_{k}}{\sum u_{i}V_{i}}=\frac{V_{k}}{bT}.$$ (5)

Hence, $\frac{v_{k}}{N}$ in Eq (4) is the initial reproductive-value-weighted frequency of the mutation [3]. In the expression in Eq (4), *σ* ^2^ is the variance in growth of the resident population. This is subject to stochastic fluctuations in size over time because individuals with identical propensities to survive and reproduce (i.e. those of the same age) may have different realized performances; i.e. demographic stochasticity [22]. For finite, density-independent, monomorphic populations that live in a constant environment, *σ* ^2^ is the demographic variance defined by Engen et al. [23] as

$$\sigma^{2}=\sum_{x=1}^{\omega }u_{x}\left[v_{1}^{2}Var\left(F_{x}\right)+v_{x+1}^{2}Var\left(p_{x}\right)+2v_{1}v_{x+1}Cov\left(F_{x},p_{x}\right)\right],$$ (6)

Where p_*k*_ is expected survival of an individual of age *k* to at least age (*k* + 1), while *F*_*k*_ is the average number of offspring produced at age *k* that are expected to survive at least to just before the next breeding season. The sum in Eq (6) excludes post-reproductive ages, because *v*_*k*_ and *F*_*k*_ are constants equal to zero when *k* > *ω*, thereby making the quantity between squared brackets equal to zero if computed at any age greater than the last reproductive age *ω*. In this model, the mutation is supposed to have a negligible effect on the resident demographic variance [3]. Note that, in Eq (4), $\frac{N}{\sigma^{2}}$ is proportional to the effective size of the age structured population, where the constant of proportionality is the reciprocal of the mean age at motherhood [24]. Varying only one parameter at the time in Eq (4), and holding all other parameters constant, Vindenes et al. [3] showed that the fixation probability of the mutation increases with the beneficial effect of the mutation and the (normalized) reproductive value of the initial mutant, while it decreases with genetic drift (i.e. greater *σ* ^2^) and initial census size. Hence, the model with age structure in Eq (4) seems to recapitulate all results from Kimura’s model. Reproductive value, which does not appear in the model without age structure, is here necessary in order to account for the fact that, depending on the age of the mutant (i.e. which may be born or migrated to the population), this may be left with a different potential for contributing to the ancestry of the future population.

## Effect of Post-Reproductive Lifespan

We now use the model with age structure to understand whether fixation is more probable in one of the two populations, *A* and *B*, described above. To this aim, we first need to understand the effect of post-reproductive lifespan on the demographic variance. We recall that, in the stationary population, the stable fraction *u*_*k*_ of individuals in age class *k* is proportional to the probability *l*_*k*_ of a newborn to survive at least to age *k*. The factor of proportionality is the birth rate *b* [25]. Therefore, using Eq (5), we can reformulate the demographic variance as

$$\sigma^{2}=\sum_{x=1}^{\omega }bl_{x}\left[{\left(\frac{V_{1}}{bT}\right)}^{2}Var\left(F_{x}\right)+{\left(\frac{V_{x+1}}{bT}\right)}^{2}Var\left(p_{x}\right)+2\frac{V_{1}}{bT}\frac{V_{x+1}}{bT}Cov\left(F_{x},p_{x}\right)\right]=b^{-1}\sum_{x=1}^{\omega }\frac{l_{x}}{T^{2}}\left[V_{1}^{2}Var\left(F_{x}\right)+V_{x+1}^{2}Var\left(p_{x}\right)+2V_{1}V_{x+1}Cov\left(F_{x},p_{x}\right)\right]=\vartheta b^{-1}.$$ (7)

Here we implicitly define the quantity *ϑ* = *bσ* ^2^. In the light of the considerations in the previous two sections, it is evident that *ϑ* is insensitive to *e*_*ω*_, because all terms in the summation in the second-line of the above expression do not refer to, or depend on, post-reproductive survival. Therefore, we can conclude that, for our populations,

$$\vartheta_{A}=\vartheta_{B}=\vartheta .$$ (8)

Suppose now that, in both populations *A* and *B*, a mutant of age *k* ≤ *ω* is introduced. Importantly, we assume that the mutation modifies in the same way the life table of both *A* and *B*, but it does not change survival at the post-reproductive stage (i.e. no modifications of *p*_*ω+k*_ with *k* ≥ 0). As *A* and *B* have the same life table up to the last reproductive age, equal changes in the shared life table imply that the difference in growth *s* between the resident population and a hypothetical population exclusively comprised by mutant individuals is equal between *A* and *B*; that is, *s*_*A*_ = *s*_*B*_ = *s* As required in [3], we assume that *s* is sufficiently small that the mutant reproductive value is approximately identical to the reproductive value of a resident individual of the same age. Using the model in Eq (4), we can derive the conditions under which fixation is more probable in *A* than in *B* by solving the inequality

$$\frac{1-\text{exp}\left\{-2s\frac{v_{k,A}}{N}\frac{N}{\sigma_{A}^{2}}\right\}}{1-\text{exp}\left\{-2s\frac{N}{\sigma_{A}^{2}}\right\}}>\frac{1-\text{exp}\left\{-2s\frac{v_{k,B}}{N}\frac{N}{\sigma_{B}^{2}}\right\}}{1-\text{exp}\left\{-2s\frac{N}{\sigma_{B}^{2}}\right\}},$$ (9)

**Fig 1:**
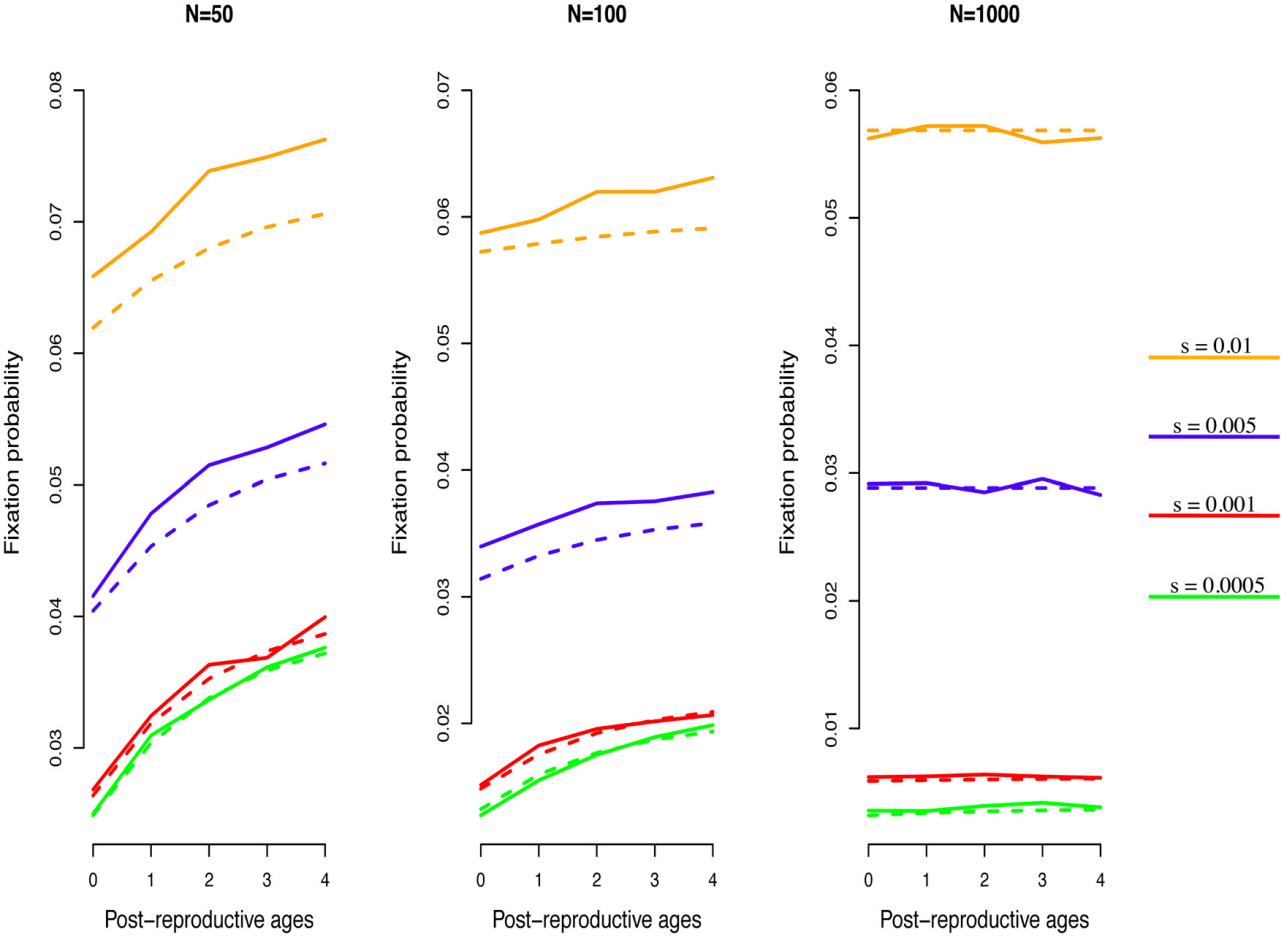
Fixation probability as a function of post-reproductive lifespan. A density-independent, stationary, closed-to-migration population of size *N* was initiated with a single mutant with advantage *s* in the first age class. At each simulation step, all individuals in age classes smaller than or equal to 2 produce exactly one newborn each. Individuals in the maximum age class (i.e. 2 + post-reproductive ages) are removed from the population. Resident and mutant individuals survive to the next age class with probability 0.618034, but while resident newborns enter the first age class with the same probability, mutant newborns do so with probability 0.618034 + *f*(s), where *f*(s)>0 is such that the mutant growth rate is equal to *s* plus the resident growth rate. The simulation run ended with population extinction or fixation of one of the two types. The population was considered extinct when there was no individual with reproductive value. Fixation for one type was considered achieved when the total reproductive value in the population was exclusively contributed to by that type’s subpopulation. Fixation probability of the mutant was calculated as the number of times it achieved fixation in 100000 simulation runs without considering those runs in which the whole population went extinct before fixation of either type. We compared simulation results (solid line) with analytic results (dashed line) derived from the approximation in Eq (4) in the main text. We explored the role of increasing the maximum attainable age, the total population size, and the magnitude of the advantageous effect of the mutation.

Where $\sigma_{A}^{2}$ and $\sigma_{B}^{2}$ are the demographic variances of *A* and *B*, respectively. Substituting from Eq (5) and Eq (7) and using Eq (2) and Eq (8), the inequality simplifies to *b*_*A*_ < *b*_*B*_, which is true under assumption Eq (1). Hence, we can predict that fixation of the beneficial mutation is more likely in the population in which life expectancy at the last reproductive age is greater, everything else being equal. Simulations are made confirming this prediction for a population closed to migration (Fig 1). A mutant can only enter this population in the first age class. We follow the method of [3] by considering a stationary, density independent population. A note on the validity of our result. Substituting from Eq (5) and Eq (7) in Eq (9), we can see that the two numerators are identical, and the difference in the fixation probability between *A* and *B* is exclusively due to the denominators in Eq (9). However, the function *f* (*x*) = 1−exp{−*x*} rapidly goes to unity. Therefore, we may expect that our prediction should hold when considering populations in which the beneficial effect and the census size are small and the demographic variance not negligible; i.e. as a rule of thumb *sN* ≤ *σ* ^2^. The result should be extendable to the sexual diploid case too under the assumptions that mating is random and vital rates are similar between females and males. The initial mutation should be found in a heterozygous individual, while all others are homozygotes for the resident allele. In this case, the diffusion approximation for the fixation probability of the mutation is identical to Eq (4) with the exception that 2*N* in the denominator should be substituted by 4*N* [3].

## Discussion

The result we have obtained concerning the relationship between post-reproductive lifespan and the computation of the fixation probability of a beneficial mutation is of interest for the relationship between models with, and models without, age structure. Take the classic model in Eq (3) for populations without age structure. In this model, *N*_*e*_ can decrease without interfering with the values of *s* and the initial frequency of the mutation $\frac{1}{N}$. Such decrease would simply indicate that genetic drift is stronger, and a beneficial mutation would be less likely to go to fixation. Extrapolating from this model to the age-structured case, one may be led to predict that prolonging post-reproductive lifespan in a population of a constant size *N* would lower the fixation chances of any beneficial mutation, because effective size gets smaller. The reason behind this intuition would be that an increase in the number of post-reproductive individuals lowers the number of individuals that breed. This amplifies the effect of random sampling of gametes through generations and, thus, the effective population size, which in an age structured population is given by $N_{e}=\frac{N}{T\sigma^{2}}$ [24], gets smaller [15]. Indeed, Vindenes et al. [3] stated that, in the model in Eq (4), “increasing the demographic variance […] always causes a decrease in the fixation probability”. However, our results show that precisely the opposite is true when effective size decreases in response to an increase in post-reproductive lifespan. Why is this so? By looking at the inequality in Eq (9) the initial reproductive-value-weighted frequency $\frac{v_{k}}{N}$ of the mutation and the effective size $\frac{N}{\sigma^{2}}$ are the only quantities that differ between the two sides of the expression. With longer post-reproductive lifespan and constant census size *N*, the former quantity increases, while the latter decreases. This is because *v*_*k*_ captures the present value of future offspring production to an individual of age *k* relative to the average reproductive value in the population. If the population comprises more post-reproductive individuals, which have zero reproductive value, then the average reproductive value gets smaller, and *v*_*k*_ gets higher for individuals that are not post-reproductive. This would tend to increase the fixation probability of the beneficial mutation. But, at the same time, the effective size of the population decreases as post-reproductive lifespan gets longer, similarly to what happens in any population in which there is a reduction in the number of individuals participating in reproduction. Such decrease would tend to make the loss of the mutation more likely because of random sampling. Therefore, a more prolonged life after the age of last reproduction has two conflicting effects on the fixation probability of the beneficial mutation. However, solving the inequality in Eq (9) shows that fixation is more likely in the population with longer post-reproductive lifespan. Evidently, the increase in the reproductive-value-weighted frequency of the initial beneficial mutation is sufficient to outweigh the effect of a decreased effective size. This result suggests that it would be wrong to infer that an increase in effective size in the age structured population necessarily leads to decreased chances of fixation for a new beneficial genetic variant. As a final note, in an infinite haploid population with age structure, the fixation probability of a beneficial mutation is $s\frac{v_{k}}{\sigma^{2}}$ [26–28]. Substituting from Eq (5) and Eq (7), this expression reduces to $s\frac{V_{k}}{\vartheta T}$, which is insensitive to post-reproductive survival. This shows that, in the infinite population, the traditional view perfectly holds according to which population genetics models are insensitive to post-reproductive lifespan [12].
